# Per-nucleus crossover covariation is regulated by chromosome organization

**DOI:** 10.1016/j.isci.2022.104115

**Published:** 2022-03-18

**Authors:** Cunxian Fan, Xiao Yang, Hui Nie, Shunxin Wang, Liangran Zhang

**Affiliations:** 1 Shandong Provincial Key Laboratory of Animal Resistance Biology, College of Life Sciences, Shandong Normal University, Jinan, Shandong 250014 China; 2 Center for Reproductive Medicine, School of Medicine, Cheeloo College of Medicine, Shandong University, Jinan, Shandong, China; 3 National Research Center for Assisted Reproductive Technology and Reproductive Genetics, Shandong University, Jinan, Shandong 250012, China; 4 Key Laboratory of Reproductive Endocrinology of Ministry of Education, Jinan, Shandong 250001, China; 5 Shandong Provincial Clinical Research Center for Reproductive Health, Jinan, Shandong 250012, China; 6 Advanced Medical Research Institute, Shandong University, Jinan, Shandong 250012, China; 7 State Key Laboratory of Microbial Technology, Shandong University, Jinan, Shandong, China

**Keywords:** Genetics, Molecular genetics, Molecular biology, Cell biology

## Abstract

Meiotic crossover (CO) recombination between homologous chromosomes regulates chromosome segregation and promotes genetic diversity. Human females have different CO patterns than males, and some of these features contribute to the high frequency of chromosome segregation errors. In this study, we show that CO covariation is transmitted to progenies without detectable selection in both human males and females. Further investigations show that chromosome pairs with longer axes tend to have stronger axis length covariation and a stronger correlation between axis length and CO number, and the consequence of these two effects would be the stronger CO covariation as observed in females. These findings reveal a previously unsuspected feature for chromosome organization: long chromosome axes are more coordinately regulated than short ones. Additionally, the stronger CO covariation may work with human female-specific CO maturation inefficiency to confer female germlines the ability to adapt to changing environments on evolution.

## Highlights

• Crossover covariation is transmitted to progenies from meiocytes without selection
• Human females have stronger crossover covariation because of longer axes
• Long chromosomes have strong correlations between axis lengths and crossovers
• Chromosomes with longer axes are more coordinately regulated

## Graphical abstract

**Figure:**
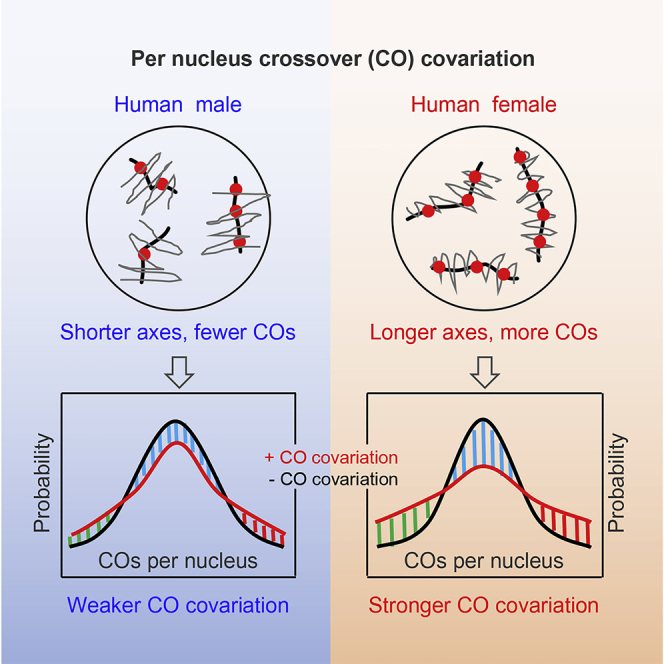

## Introduction

Meiotic crossover (CO) recombination between homologous chromosomes (homologs) is required for faithful chromosome segregation and promotes genetic diversity by shuffling alleles along chromosomes (Bell, 1982; Veller et al., 2019). A high frequency of chromosome segregation errors occurs in human female meiosis, which is a major cause of aneuploid conceptions, resulting in infertility, abortion, and congenital disorders (Nagaoka et al., 2012). Studies show that females have different CO recombination features compared with males and at least some of these features promote the formation of aberrant CO configurations and thus aneuploidy (Nagaoka et al., 2012; Gruhn et al., 2013; Ottolini et al., 2015; Wang et al., 2017; Hassold et al., 2021). An important one is the female-specific CO maturation inefficiency, which results in a fraction of designated CO sites failing to become actual COs (Figures S1A and S1B) (Wang et al., 2017). As a consequence, a high frequency of chromosomes with vulnerable CO configurations (especially chromosomes that have no or only distal localized COs), which are subject to missegregation (Wang et al., 2021a).

The formation of CO recombination is integrated into and thus tightly regulated by meiotic chromosomes, which are organized as linear arrays of loops on the proteinaceous axes (Figure S1A) (Kleckner, 2006; Wang et al., 2021a).

One central feature of meiotic COs is that axis length, which is determined independent of recombination, is the basic determinant of CO number (Kleckner et al., 2003; Zhang et al., 2014b; Wang et al., 2019c, 2021a). A general positive correlation between chromosome axis length and CO frequency leads to the hypothesis that variations in axis length are a major contributor to CO variation (Kleckner et al., 2003). Accumulated evidence supports this general idea: alterations in chromosomes axis length always result in coordinate alterations in CO frequency; however, alterations in DSBs and/or DSB-mediated recombination intermediates do not change axis length and CO formation only have subtle effects on local chromosome axis length (reviewed in Wang et al., 2021a; Shang et al., 2022). Consistently, human female meiosis has a longer chromosome axis and consequently more COs than male meiosis (Figure S1B) (e.g., Bojko, 1985; Lynn et al., 2004; Tease and Hultén, 2004; Codina-Pascual et al., 2006; Gruhn et al., 2013; Wang et al., 2017, 2019c). Recently, we have shown that gradual alterations in axis lengths result in coordinate and gradual alterations in the numbers of meiotic DSBs and COs (Song et al., 2021; Wang et al., 2021b). The axis length also regulates CO distributions along chromosomes, for example, COs tend to be more distally located on short chromosomes (Wang et al., 2021a; Shang et al., 2022). However, how chromosome axis length is regulated is unclear. It is worth noting that genetic factors affecting only SC length or only CO number have also been suggested (e.g., Vranis et al., 2010; Wang et al., 2012, 2019a; Sandor et al., 2012; Ziolkowski et al., 2017). These factors may disturb the tight correlation between the axis length and CO number.

**Figure 1:**
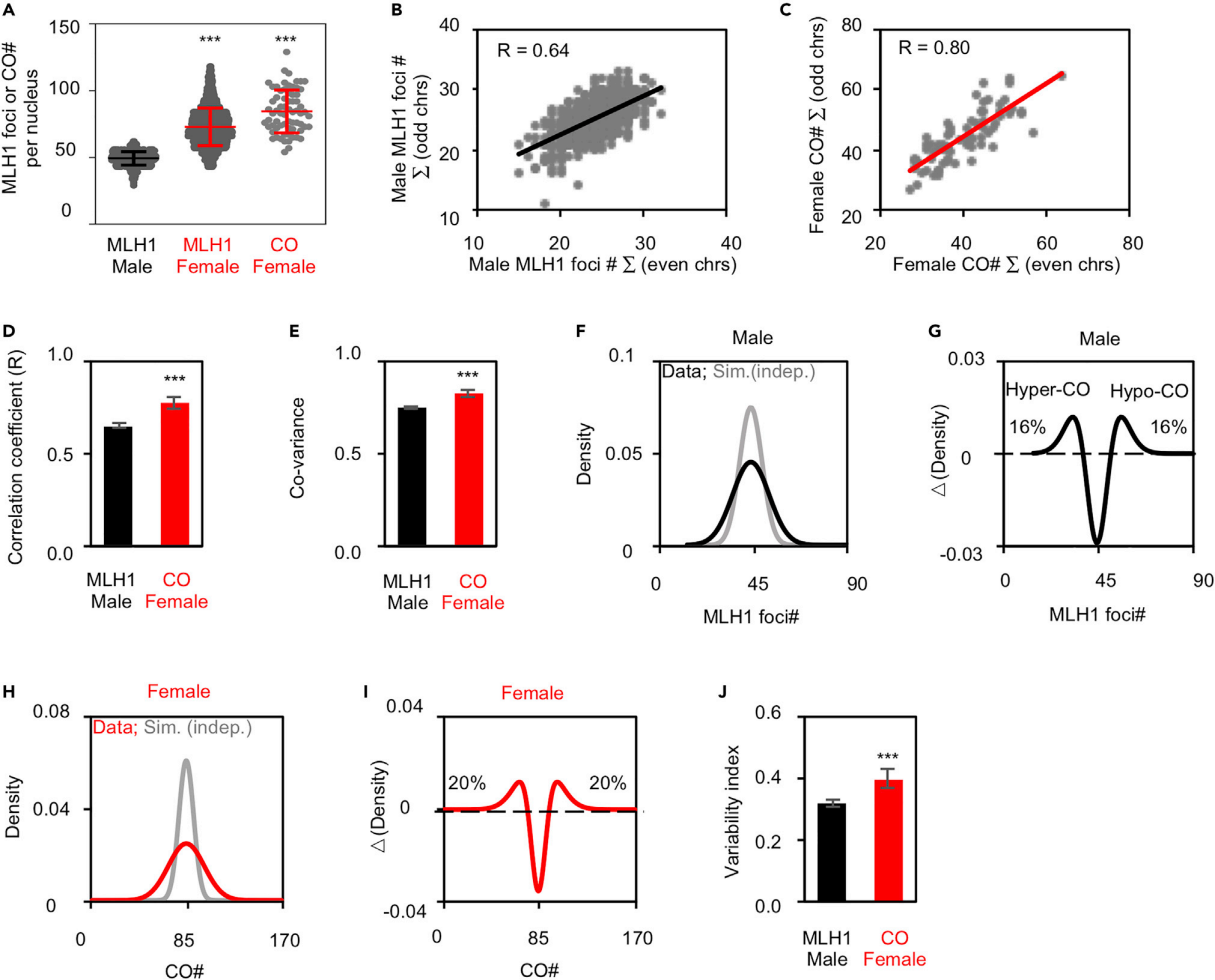
Analysis of CO covariation at per-nucleus level (A) Scatter plot of the nucleus number of CO-associated MLH1 foci or COs from sequencing. (B) Scatter plot to show the correlation for the numbers of MLH1 foci between odd and even chromosomes from spermatocytes. R, Pearson’s correlation coefficient. (C) Scatter plot to show the correlation for the numbers of COs between odd and even chromosomes by sequencing of female “tetrads.” R, Pearson’s correlation coefficient. (D) Correlation coefficient between two matched groups of chromosomes for male MLH1 foci and female COs. Chromosomes were divided into two comparable groups with each group hearing half number of all chromosomes (or one group contains one more chromosome than the other if there is an odd number of chromosomes). Correlation coefficients were calculated for all matched groups of chromosomes. (E) CO-variance for male MLH1 foci and female COs. (F) The best-fit curves for distributions of the experimental (data; black curve) and simulated (sim.; gray curve) MLH1 number per nucleus. The simulation was conducted under the assumption that MLH1 foci are independent of different chromosomes within nuclei. (G) The fractions of nuclei with hypo- and hyper-COs by subtracting the gray curve from the black curve in (F). (H) As in (F) but calculated from female COs. (I) As in (G) but calculated from (H). (J) Viability index (the sum of the fractions of nuclei with hyper- and hypo-COs). Samples: N = 755 spermatocytes for males and 69 “tetrads” (egg and two polar bodies) for females (A–J); female MLH1 foci from 2078 oocytes were kindly provided by Terry Hassold (A); 31228 (sperm), 69 (eggs of the 69 female tetrads). Panels A, B, D, and E, re-plotted with the data from Figures 1B and 2A in Wang et al. (2019c). Data are presented as mean ± SD (A) or mean ± SE (D, E, and J). ∗∗∗, p < 0.001 by Mann-Whitney test (A) or *t*-test (D, E, and J). See also Figures S1–S3 and Table S1.

A second central feature of meiotic CO is that CO levels covary across chromosomes within each nucleus. The underlying effect is analogous covariation in chromosome axis lengths (Figure S1C) (Wang et al., 2019c). The per-nucleus CO covariation increases the differences in CO numbers among nuclei and thus gives rise to higher frequencies of nuclei (thus gametes) with hypo- and hyper-COs, providing an evolutionary advantage in changing environments (Wang et al., 2019b, 2019c). Interestingly, human females have stronger CO covariation than males; however, the reason for this difference is unknown (Figure 1).

We are interested in further investigating the less studied CO covariation and discovering possible new features for CO regulation by axes using available human data. Here, we demonstrate that human females have stronger CO covariation than males. CO covariation is transmitted to progenies from meiocytes without selection in both human males and females. Further investigations show that chromosomes with longer axes tend to have stronger axis length covariation and a stronger correlation between axis length and CO number, and consequently stronger CO covariation. This suggests the stronger per-nucleus CO covariation in human females probably arises from the longer chromosome axis. Stronger CO covariation in females generates larger fractions of nuclei with fewer (and more) COs. CO maturation inefficiency preferentially affects nuclei with fewer COs (designation sites) and makes them more vulnerable to chromosome missegregation. These findings reveal a previously unsuspected genetically determined feature in chromosome organization: chromosomes with longer axes tend to vary and be modulated more coordinately at a per-nucleus level.

## Results

### The strength of CO covariation is determined by the strength of CO correlation

In humans, COs can be defined by the analysis of genotype data based on DNA polymorphisms or the analysis of chiasmata or CO-associated MLH1 foci (Figure 1A) (e.g., Wang et al., 2017; Hassold et al., 2021). Recently, an investigation of CO numbers at the per-chromosome and per-nucleus levels revealed a new feature of meiotic recombination in various organisms, including human males and females—CO covariation. If one bivalent (a pair of homologs comprising four chromatids) has relatively more (fewer) COs, each bivalent within the nucleus tends to have more (fewer) COs, that is, the numbers of COs on different bivalents tend to vary coordinately (Wang et al., 2019c). This phenomenon, CO covariation, can be directly determined by the CO correlation coefficient (R) between two comparable chromosome groups (e.g., odd-numbered and even-numbered chromosomes; Figures 1B–1D; STAR Methods). CO covariation can also be reflected by the contribution of CO covariance to the total variance, that is, normalized CO covariance (Figures 1E and S2; STAR Methods), and the CO variability index, which is the sum of the proportions of nuclei with more (hyper) and fewer (hypo) COs than that without CO covariation (COs on chromosomes are independent of each other within nuclei) (Figures 1F–1J; STAR Methods).

**Figure 2:**
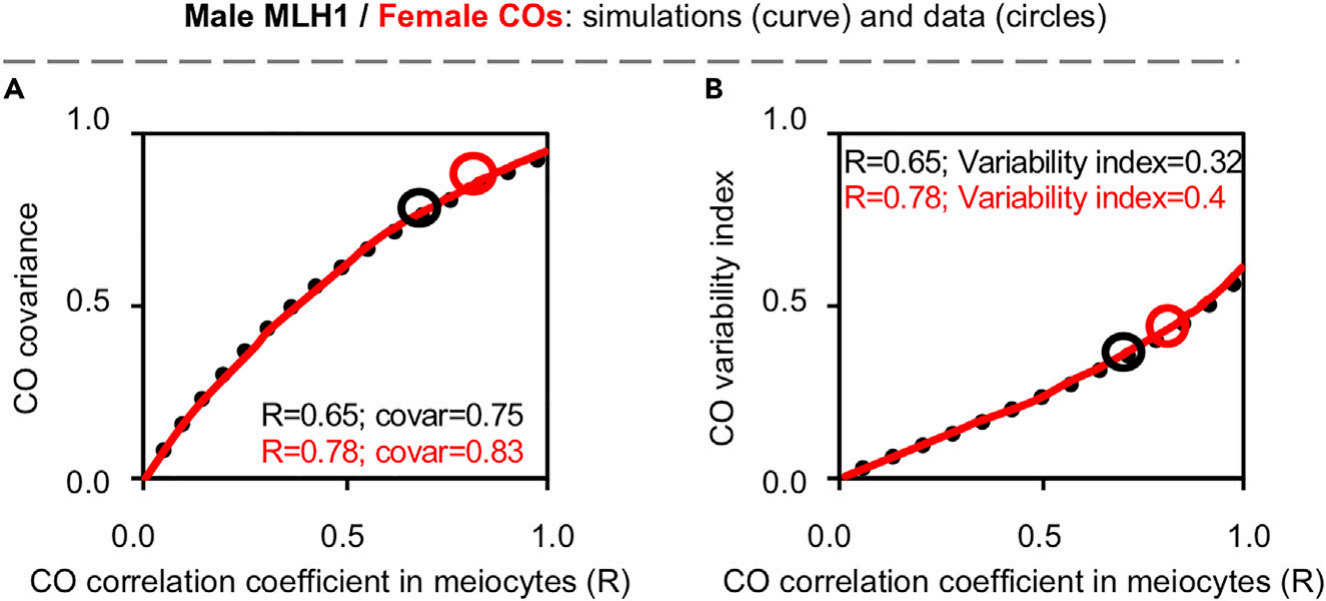
The CO correlation strength determines CO covariation strength and variability index in meiotic nuclei Bivalents in spermatocytes or female “tetrads” were pooled and reordered according to the number of COs on that bivalent, separately, for each chromosome. A series of sets of “in silico” nuclei with different CO correlation coefficients were created as described in STAR Methods. (A and B) The CO covariance (A) and variability index (B) calculated from each set of the “in silico” nuclei were plotted against the corresponding CO correlation coefficient to generate covariance curves (A) and variability index curves (B). Black dot curve, based on male MLH1 focus data; red curve, based on female COs. Black circle, calculated from male MLH1 data; red circle, calculated from female CO data. The numbers of spermatocytes and female “tetrads” as in Figure 1. See also Figure S4 and Table S1.

To quantify how the CO correlation coefficient affects CO covariance and variability index, the bivalents analyzed in spermatocytes or female “tetrads” (the egg and the two polar bodies of an individual oocyte) were pooled and reordered according to the number of COs on that bivalent, separately, for each chromosome. One bivalent from each chromosome was orderly taken and put into an artificial nucleus, which resulted in a set of artificial nuclei “in silico,” with the strongest CO correlation between chromosomes. The strength of the CO correlation was gradually disturbed from this set of nuclei to create a series of sets of “in silico” nuclei with a gradually decreased CO correlation coefficient (STAR Methods). The CO covariance and variability index were calculated from each set of the “in silico” nuclei and plotted against the corresponding CO correlation coefficient. A stronger CO correlation generates stronger CO covariance and variability index (Figure 2). Although human females have ∼1.5-fold more COs than males (Figure 1A), the same strength of CO correlation gave the same strength of CO covariance and variability index (Figure 2, solid and dotted curves). Mathematically, for two variables *X* and *Y* with standard deviations σX and σY, the relationship between correlation coefficient (corrXY) and covariance (covΧY) is defined as: corrXY=covXY/(σXσY). Thus, the above result suggests that the strength of the CO correlation probably determines the CO covariance and variability index levels, and females have stronger CO covariance and a larger variability index probably because females have a stronger CO correlation (Figure 2, black and red circles).

### CO covariation is transmitted to progenies from meiocytes without detectable selection

**Figure 3:**
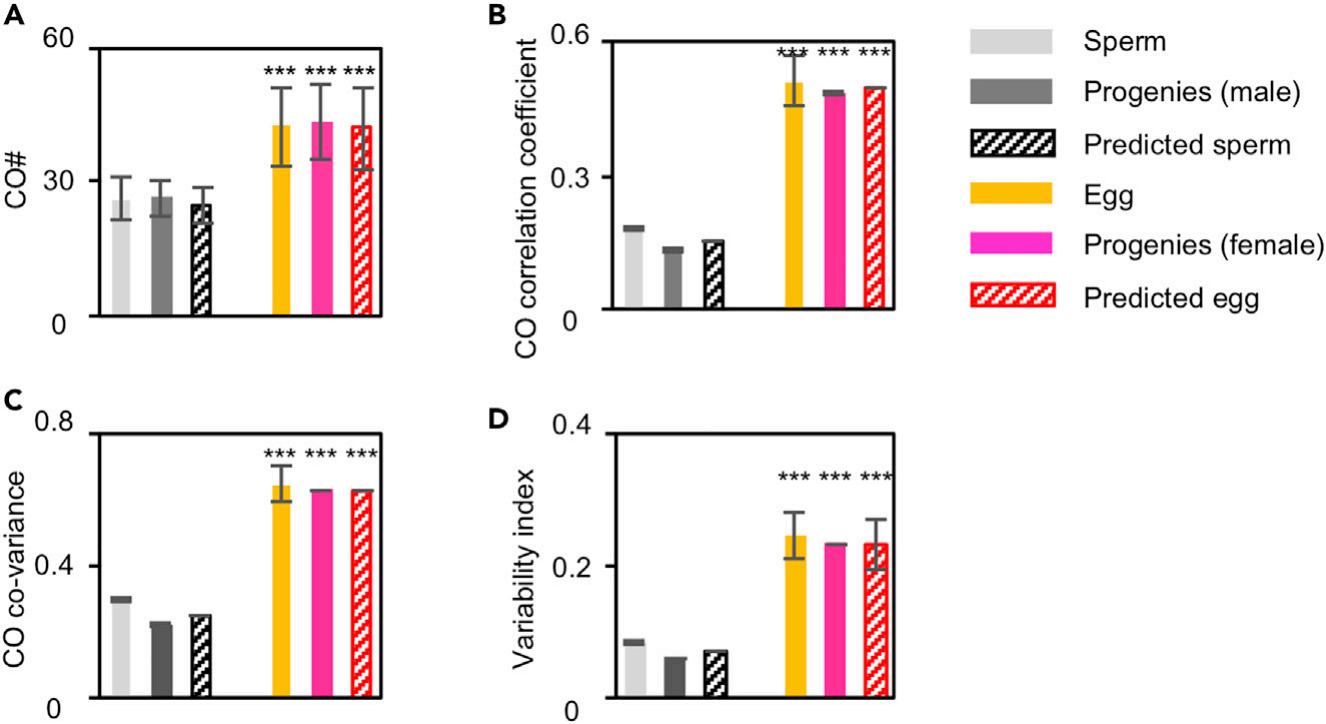
Stronger CO covariation in human female gametes and progenies than in males (A) CO number in gametes and progenies. (B) CO correlation coefficient between two matched groups of chromosomes in gametes and progenies. (C) CO co-variance in gametes and progenies. (D) CO variability index in gametes and progenies. Samples: N = 755 spermatocytes from males and 69 “tetrads” (egg and two polar bodies) from females were used to calculate predicted gametes, 31,228 sperm, 69 eggs (from the 69 female tetrads), 56321 male progenies, and 70086 female progenies. Data are presented as mean ± SD (A) or mean ± SE (B-D). ∗∗∗, p < 0.001 by *t*-test (A–D). See also Tables S2 and S3.

CO covariation has been proposed to have an important role in the evolutionary adaption by concomitantly generating more fractions of nuclei with more and fewer COs (Wang et al., 2019c). This evolutionary benefit requires CO covariation to be transmitted to progenies. Each CO forms between two non-sister chromatids. The existence of one CO on a pair of homologs reduces the probability of the occurrence of another CO nearby, which is the phenomenon of CO interference (Jones and Franklin, 2006; Zhang et al., 2014a). Meanwhile, the existed CO may affect the usage of chromatids for another CO, for example, the second CO may tend to use the other two non-sisters not used by the first CO. This phenomenon is called chromatid interference. Studies suggest that there is actually no chromatid interference (e.g., Zhao et al., 1995; Wang et al., 2021a). If so, each CO on a bivalent occurs on two non-sister chromatids by chance. As a result, each CO has a 50% possibility of being transmitted to a given gamete, and each gamete receives only half of the COs from the meiotic nucleus (compare Figure 3A with 1A). This would decrease the CO correlation coefficient and consequently covariance and variability index in gametes/progenies.

Previously, with a limited number of sperm/eggs, CO covariation in meiocytes was found to be transmitted to gametes (Wang et al., 2019c). Recently, CO frequencies were measured on each chromosome from a large number of sperm and progenies (men and women) by whole-genome resequencing (Halldorsson et al., 2019; Bell et al., 2020). Taking advantage of these data, we measured the CO correlation coefficient and also CO covariance and variability index in sperm and progenies. As expected, their values were all decreased compared with that in meiocytes (spermatocytes/oocytes) (compare Figures 3B–3D with 1D, 1E, 1J, respectively). More importantly, comparable values were obtained for each of these parameters, calculated from gametes, progenies, and predicted gametes from meiocytes under the assumption of no chromatid interference for males and females, respectively (Figures 3B–3D). Therefore, per-nucleus CO covariation can be transmitted to gametes and then to progenies without detectable selection.

### Stronger CO covariation in human females than males

As in many other organisms, human meiotic recombination shows sexual dimorphism (e.g., Gruhn et al., 2013; Wang et al., 2017). Compared with human males, females have longer chromosome axes and thus more COs (Figure 1A) (Bojko, 1985; Lynn et al., 2002; Kleckner et al., 2003; Tease and Hultén, 2004; Gruhn et al., 2013; Wang et al., 2017, 2019c). In addition, females are associated with the unique CO maturation inefficiency (Wang et al., 2017, 2021a). Per-nucleus CO covariation exists in both human males and females (Figure 1) (Wang et al., 2019c). However, compared with human males, females have stronger CO covariation as revealed by a stronger CO correlation coefficient (Figure 1D), accompanied by a stronger CO covariance (Figures 1E and S2) and a higher CO variability index (Figure 1J).

There are two types of COs in most organisms (Hunter, 2015; Zhang et al., 2014a; Gray and Cohen, 2016; Pazhayam et al., 2021; von Diezmann and Rog, 2021). Type I COs are marked by MLH1 foci at pachytene and interference-insentive and their number and distribution are tightly controlled. However, Type II COs are interference-sensitive and randomly distributed on chromosomes. In humans, Type II COs are estimated to be ∼10% of total COs (Housworth and Stahl, 2003; Lenzi et al., 2005; Hou et al., 2013; Cheng et al., 2009). To validate our above comparisons between males (MLH1 foci marked Type I COs) and females (total COs from DNA sequencing) and to evaluate the influence of Type II COs on CO covariation, a total CO data set (including Type I and II COs) was generated by randomly adding ∼10% of COs to each chromosome of human spermatocyte MLH1 COs (STAR Methods). The strength of covariation for total COs was calculated based on this new CO dataset. Randomly distributed Type II COs would decrease CO correlation strength. As expected, the covariation level for total COs was lower than that for MLH1 COs (Figure S3). This result validates our above (and also below) conclusions based on CO covariation comparisons between human males and females and implies the actual difference would be larger if female MLH1 CO data (or male total CO data) were used for comparisons. It is also worth to note that there is increased variability in the recombination rate in females compared with males (e.g., Broman et al., 1998; Kong et al., 2002; Wang et al., 2017). This raises the possibility that the greater CO correlation may result from the greater variation. However, this is not the case as the greater CO variation requires CO maturation inefficiency as previously revealed (see Figure 5B middle in ref. Wang et al., 2017), which actually decreases but does not increases CO correlation strength (Figure S3).

Stronger CO covariation was also observed in female than male gametes/progenies (Figure 3, compare females vs males). The differences in CO covariation between male and female gametes/progenies were significantly enlarged compared to those in meiocytes, because the degree of reduction in females was relatively moderate (compare Figures 3B–3D with 1B, 1E, and 1J). For example, from meiocytes to gametes/progenies, the CO correlation coefficient decreased from 0.65 to 0.29 (∼55% reduction) in males; however, it decreased only from 0.78 to 0.64 in females (∼18% reduction) (compare Figure 1D with 3B). This result was also confirmed by accurately mathematical calculations described in “covariance analysis” in the section of STAR Methods. As in meiocytes, the strength of CO covariation was probably also determined by the strength of CO correlation as the same strength of CO correlation gave the same levels of CO covariance and variability index in gametes/progenies (Figures 2 and S4).

**Figure 4:**
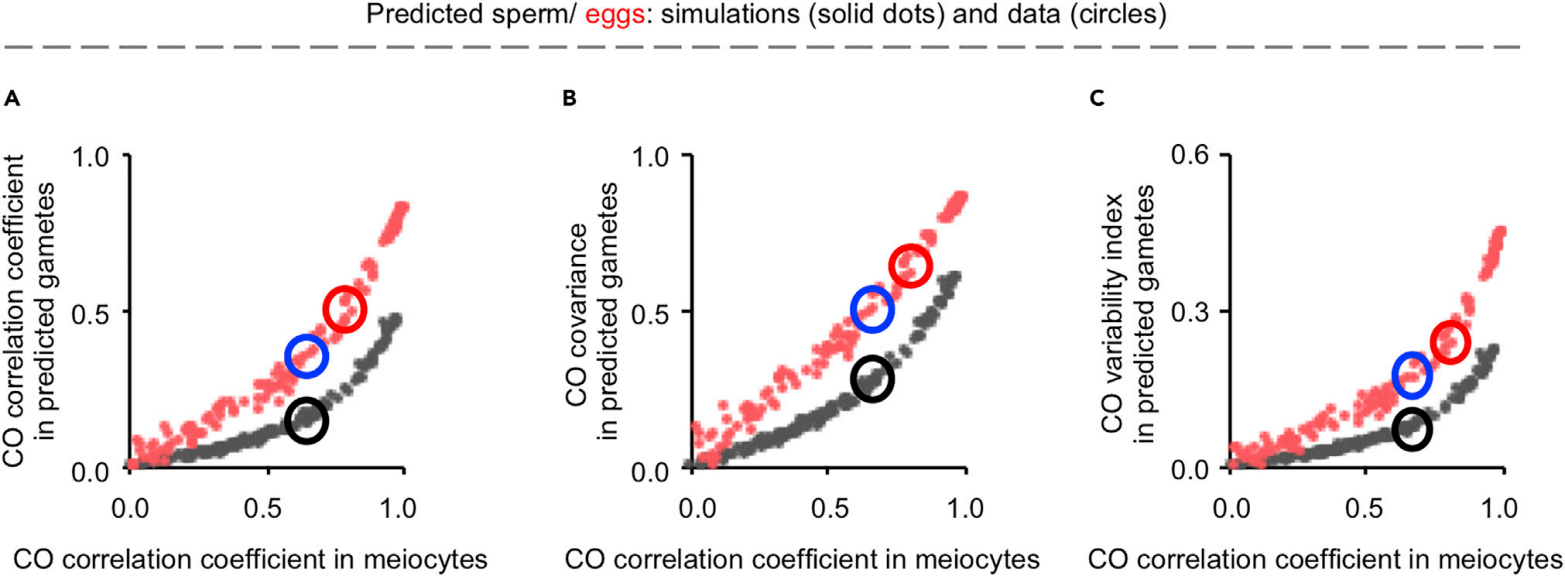
CO covariation altered differently from meiocytes to gametes in human males and females A series of sets of “in silico” nuclei with different CO correlation coefficients were created based on male MLH1 foci or female COs as described in STAR Methods. For each set of “in silico” nuclei, a set of predicted gametes were calculated assuming no chromatid interference. The CO correlation coefficients were calculated from each set of “in silico” nuclei and the corresponding set of predicted gametes. The CO covariance and variability index were also calculated from each set of predicted gametes. (A) CO correlation coefficient in predicted gametes plotted against the corresponding CO correlation coefficient “in silico” nuclei. (B) CO covariance in predicted gametes plotted against the corresponding CO correlation coefficient “in silico” nuclei. (C) CO variability index in predicted gametes plotted against the corresponding CO correlation coefficient “in silico” nuclei. The black and red circles, corresponding values for male and female gametes, respectively. The blue circles, values of predicted female gametes from meiocytes with male (but not female) CO correlation coefficient. See also Figures S4 and S5.

To understand how CO correlation strength changes from meiocytes to gametes/progenies, we calculated CO correlation and also CO covariance and variability index from predicted gametes. At different CO correlation strengths, the values for these parameters obtained from predicted gametes and actual gametes/progenies matched each other (Figure S5). Interestingly, at the same level of CO correlation coefficient in meiocytes, a stronger CO correlation and consequently higher CO covariance and variability index levels were observed in female than male gametes/progenies (Figure 4). This difference probably results from the fact that human females have more COs than males (see discussion). Therefore, female gametes/progenies have stronger CO covariation probably because oocytes have a stronger CO correlation and more COs.

### Long chromosome pairs tend to have strong axis length correlations

**Figure 5:**
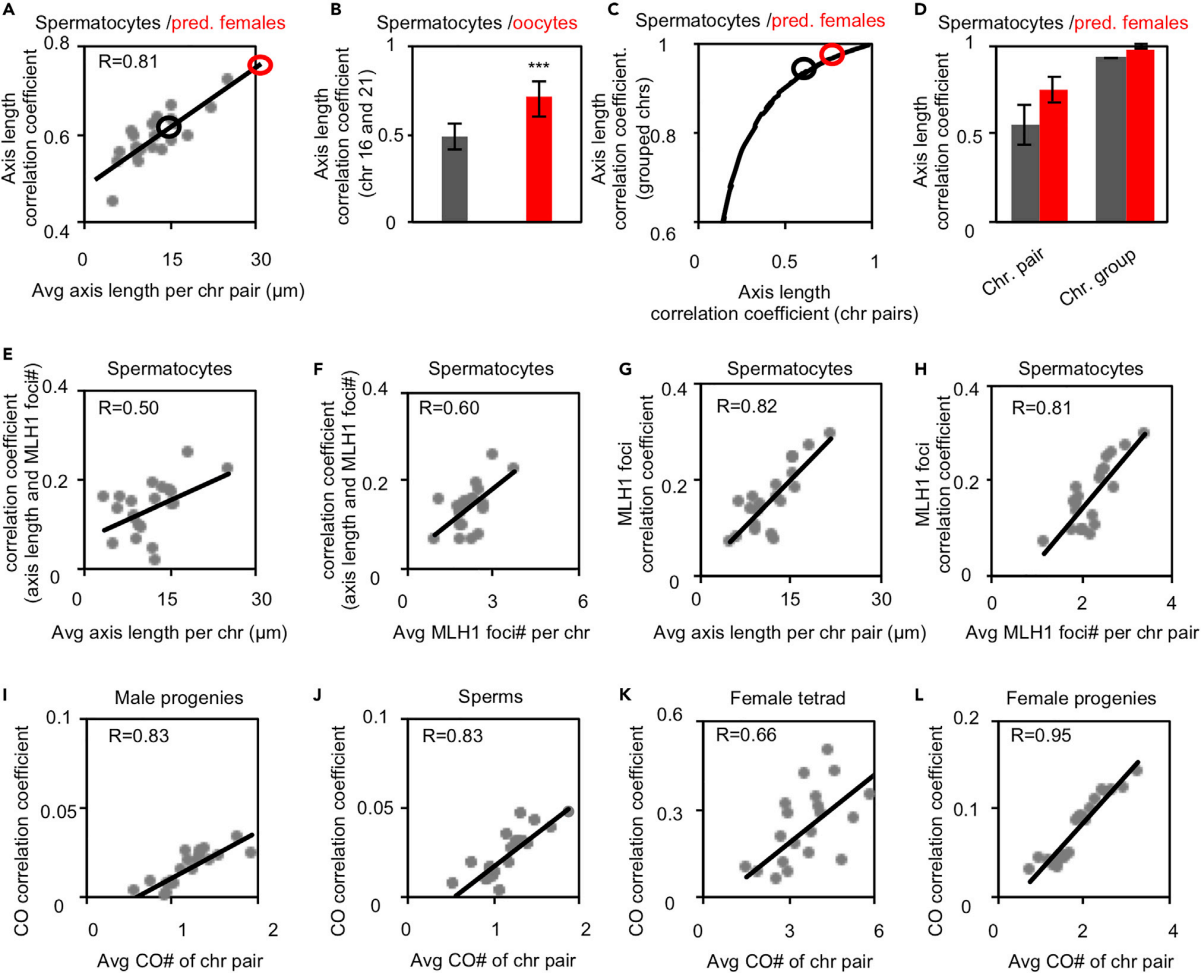
Chromosome pairs with longer axes tend to have stronger CO correlation and axis length correlation Chromosomes were ordered according to CO number or axis length, and the correlation coefficient was calculated between each pair of adjacent chromosomes. (A) Axis length correlation coefficient plotted against corresponding average axis length. The chromosome pair with longer axes tends to have a stronger correlation coefficient for axis length. In human males, the average axis length per pair of chromosomes is ∼15 μm at pachytene (e.g., Wang et al., 2017), which is corresponding to the average axis length correlation coefficient of ∼0.6 (black circle). If the same tendency exists in females, that is, axis correlation strength is determined by the axis length, the correlation coefficient of the axis lengths between chromosome pairs is predicted to be 0.75 (red circle). (B) The axis length correlation coefficient between chromosomes 16 and 21 in males (black) and females (red). Error bar, 95% confidence interval from bootstrapping. (C) The correspondence for the axis length correlation coefficient between individual chromosome pairs to that between two matched chromosome groups based on male MLH1 data. Black circle, corresponding to male MLH1; red circle, the predicted value for females. (D) The axis length correlation coefficient between individual chromosome pairs or between two matched chromosome groups in males (black bars), and the predicted values based on axis length in females (red bars). Error bar, SE. (E and F) Correlation coefficients between the axis length and the corresponding number of MLH1 foci plotted against either the axis length (E) or MLH1 foci number (F). (G and H) Correlation coefficients for MLH1 foci between adjacent pairs of chromosomes plotted against either the corresponding average axis length (G) or MLH1 focus number (H). (I–L) CO correlation coefficient plotted against the corresponding average CO number of chromosome pair for male progenies (I), sperm (J), female tetrads (K), and female progenies (L). The numbers of nuclei, gametes, and progenies as in Figures 1 and 3. R, Pearson’s correlation coefficient (A and E–L). Data are presented as mean ±95% confidence interval (B) or mean ± SE (D). ∗∗∗, p < 0.001 by *t*-test (B, D). See also Figures S6–S8 and Tables S1, S2, and S3.

Chromosome axis length covariation contributes to CO covariation (Wang et al., 2019c). The stronger CO covariation in human females probably indicates stronger axis length covariation in females than males. If the strength of axis length covariation is determined by an intrinsic property of chromosomes, more specifically, axis length *per se*, stronger axis length covariation would be observed for chromosome pairs with longer axis lengths in human males. To test this idea, chromosomes were ordered from small to large according to the axis lengths, and each chromosome was paired with its larger neighboring chromosome (STAR Methods). The axis length correlation coefficient of each chromosome pair was calculated and plotted against the corresponding average axis length (Figure 5A). By doing so, a new feature of the regulation of chromosome organization emerged: chromosome pairs with longer axes tended to have stronger axis length correlations (Figure 5A). This result was further confirmed by the same analysis with all possible pairs of chromosomes (Figure S6A). Importantly, the stronger axis length covariation is not caused by larger axis length variation on long chromosomes as all chromosomes have a similar coefficient of variation (CV) (Figure S6B). This observation suggests that the organization of long chromosomes is regulated more coordinately than short chromosomes.

The absence of chromosome axis length data from human females, which can be used for the same analysis as in males (multiple chromosomes examined in the same nucleus), prevents us from directly verifying the possibility that human females have stronger axis length covariation than males. Fortunately, we had the data for a pair of chromosomes, chromosomes 16 and 21. The average axis length of this chromosome pair is 15 μm in females and 7 μm in males. The axis length correlation coefficient for this chromosome pair is 0.72 in females and, as expected, it is much larger than that in males, which is only 0.48 (Figure 5B). Moreover, the axis length correlation coefficient (0.72) in females was slightly larger than correlation coefficients for chromosome pairs with similar axis lengths in males (Figure S6A). These results suggest that the axis length correlation coefficient in females is larger than that in males for a given chromosome pair, and also larger than or at least comparable with that in males for chromosome pairs with similar axis length. Therefore, the strength of axis length correlation is probably determined by the axis length.

As covariation is directly determined by the correlation between two grouped chromosomes, the axis length correlation coefficients from individual chromosome pairs and grouped chromosomes were compared with male data (Figure 5C). In human males, the average axis length correlation coefficient of 0.6 between individual chromosomes (Figure 5A, black circle; 5D left, black bar), which corresponded to the correlation coefficient of 0.93 between grouped chromosomes (Figure 5C, black circle; Figure 5D right, black bar). In human females, the predicted correlation coefficient between individual chromosomes was 0.75 given that the female axis length is approximately 2-fold the male axis length (Figure 5A red circle, 5D left, red bar) (e.g., Wang et al., 2017), and the corresponding correlation coefficient between grouped chromosomes was 0.97, assuming that the same relationship exists in females and males (Figure 5C, red circle; Figure 5D right, red circle). These analyses suggest that human females probably have a stronger axis length correlation arising from longer axes.

### Long chromosomes tend to have strong correlations between axis lengths and COs

Accumulative evidence suggests CO number is largely determined by the axis length (introduction). CO covariation requires not only axis length covariation but also a correlation between axis length and CO number (Figure S7). In human males, chromosomes with long axes tended to have a strong correlation between axis length and CO number (Figure 5E). This result, in combination with the fact that long chromosomes have more COs, would predict that chromosome pairs with more COs tend to have a strong correlation between axis length and CO number. This prediction was also confirmed (Figure 5F). These results indicate that females have a stronger CO and axis length correlation probably because females have longer axes and more COs than males.

Longer chromosomes have more COs, stronger axis length correlations, and stronger correlations between axis lengths and CO numbers. These results lead to two further predictions: (1) long chromosome pairs tend to have strong CO correlations, which was observed in human males (Figure 5G); and (2) chromosome pairs with more COs also tend to have stronger CO correlations. The second prediction was also confirmed in both human males and females with data from both meiocytes and gametes/progenies by the analog analysis (Figures 5H–5L). The result that there was stronger CO correlation between individual chromosomes with more COs was further confirmed by the same analysis conducted with all pairs of chromosomes (Figure S6C). Moreover, it was not owing to larger CVs for CO number on chromosomes with more COs (Figure S6D).

Further comparisons showed that for chromosome pairs with a similar number of COs in males and females, the CO correlation in males was stronger than that in females, for example, for chromosome pairs with an average of 3 COs, the CO correlation coefficient was 0.26 in males but only 0.18 in females (compare Figure 5H vs 5K). This observation was probably caused by the female-specific CO maturation inefficiency, which randomly eliminates a fraction of CO intermediates and decreases the correlation between the matured CO number and axis length. However, for a given chromosome pair, a stronger CO correlation was observed in females than males because females have much longer axes and more COs than males (Figures 5H and 5K). The same tendency was also observed in male and female gametes/progenies (Figures 5I, 5J, and 5L).

The number of COs in female progenies increased slightly with increased maternal age (e.g., Coop et al., 2008; Campbell et al., 2015; Halldorsson et al., 2019). However, this small increase in CO number has little effect on CO covariation (Figure S2).

Early studies support that chromosome axis length largely determines CO frequency (introduction). Our current analyses further support that axis length also determines axis length correlation, CO/axis length correlation, and ultimately CO correlation. Human females have longer axes and more COs and thus presumably stronger axis length covariation and stronger correlation between axis lengths and COs, and these two effects would work together to ultimately result in stronger CO covariation than that in males.

### Quantitative simulations support that females have stronger CO covariation because of longer chromosome axes

The above analyses suggest that human females have longer chromosome axes and thus stronger CO covariation. Previously, we developed a method to quantitatively simulate and analyze CO patterns in various conditions, including the effects of chromosome axis length on CO patterns (Kleckner et al., 2004; Zhang et al., 2014a; White et al., 2017). This method can accurately describe meiotic CO patterns in various organisms, including males and females (Zhang et al., 2014a, 2014b), and can help to identify new meiotic recombination features, including CO maturation inefficiency, CO covariation, recombination differences between the two sexes, and the first CO interference pathway (Zhang et al., 2014b; Wang et al., 2017, 2019c; Luo et al., 2019; Lloyd and Jenczewski, 2019).

**Figure 6:**
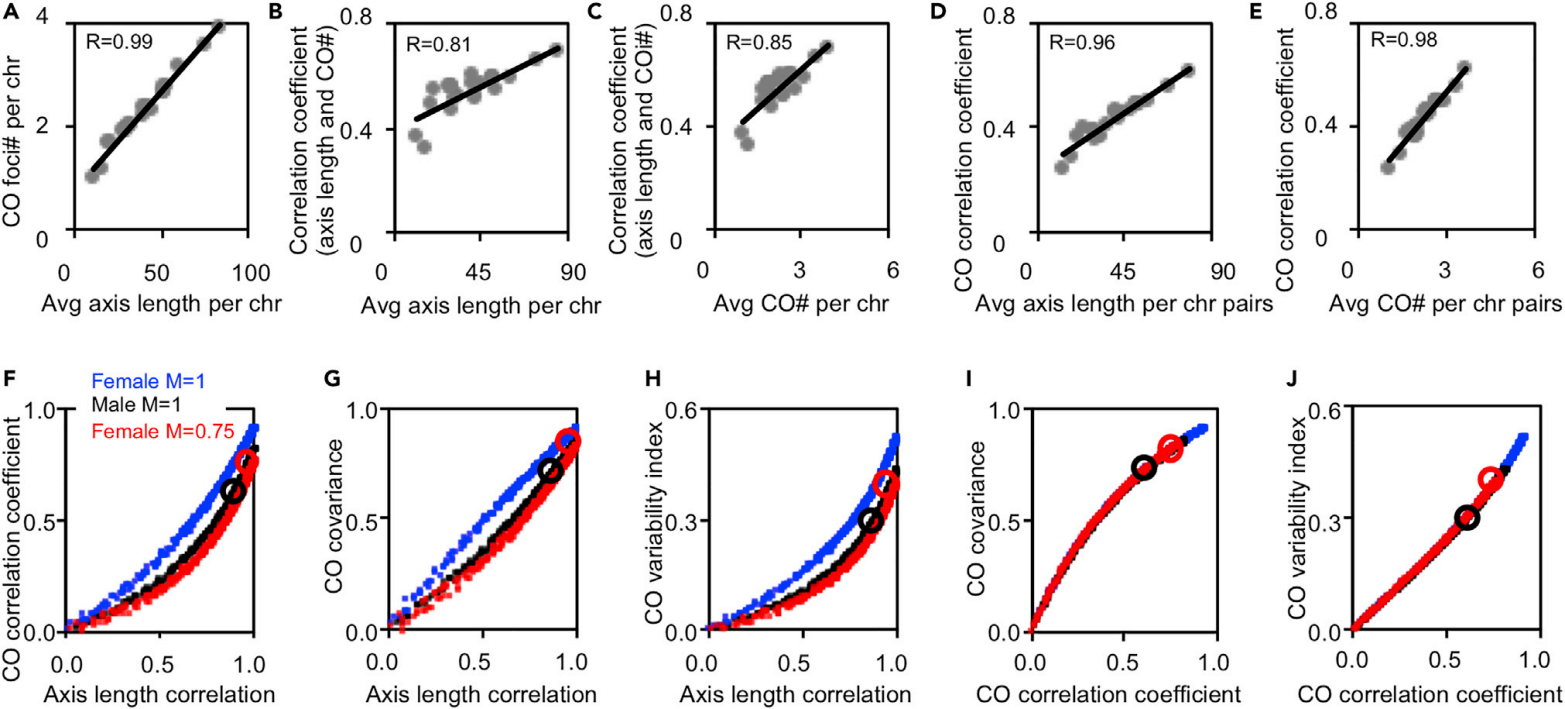
Quantitative simulations support that the axis length regulates CO covariation. MLH1-associated COs on each chromosome in spermatocytes and oocytes are quantitatively simulated (A) Simulated MLH1-associated CO number plotted against the corresponding axis length for males. (B and C) The correlation coefficient between the axis length and MLH1-associated CO number plotted against the axis length (B) or MLH1 focus number (C). (D and E) The correlation coefficient for MLH1-associated CO number between adjacent chromosomes plotted against the average axis length (D) or MLH1 focus number (E) for this pair of chromosomes. (F–H) As in Figure 2, but using simulated bivalents in males and females (with and without maturation inefficiency), to generate the curves for CO correlation coefficient (F), CO covariance (G), and variability index (H) against axis length correlation. Black circle, calculated from the best-fit simulation of male MLH1; red circle, calculated from the best-fit simulation of female MLH1. (I and J) As in (G and H), but plotted against CO correlation coefficient to show correlation strength determines CO covariance (I) and CO variability index (J). Black circle, calculated from the best-fit simulation of male MLH1; red circle, calculated from the best-fit simulation of female MLH1. R, Pearson’s correlation coefficient (A–E). See also Figures S6–S8.

Here, this simulation was performed exactly as previously described to further investigate the regulation of CO covariation (STAR Methods) (Wang et al., 2019c). Three types of parameters need to be specified: (1) the number (which also represents the axis length) and distribution of CO precursors on each bivalent, (2) CO designation driving force and CO interference strength (the distance over which the interference signal spreads), and (3) CO maturation efficiency. CO correlation coefficient, covariance, and variability index in both human males and females (with and without CO maturation inefficiency) were calculated under different axis length correlation levels (Figure 6).

These simulations confirmed and extended the above results and conclusions. (1) Chromosomes with longer axes tended to have more COs and stronger correlations between CO number and axis length (Figures 6A–6C). (2) Chromosome pairs with longer axes (more COs) tended to have stronger CO correlations (Figures 6D and 6E). (3) CO correlation coefficient, covariance, and variability index all increased along with increased axis length correlation strength in both males and females (Figures 6F–6H). (4) At the same axis length correlation level, chromosomes with longer axes (in females) have stronger CO correlation, covariance, and variability index (Figures 6F–6H, compare blue vs black). (5) CO maturation inefficiency decreases CO correlation, covariance, and variability index (Figures 6F–6H, compare red vs blue). (6) Regardless of the axis length difference and CO maturation inefficiency, the same CO correlation strength results in the same level of CO covariance and variability index (Figures 6I and 6J). Therefore, the stronger CO covariation in human females probably results from the longer chromosome axes (which show stronger axis length covariation and a stronger correlation between axis length and CO number).

## Discussion

The per-nucleus CO covariation is transmitted to progenies in human males and females without selection. Further investigations reveal an unexpected feature of chromosome loop/axis organization: chromosomes with longer axes vary more coordinately. Moreover, chromosomes with longer axes have a stronger correlation between axis lengths and CO numbers. These two features work together to cause stronger CO covariation for chromosome pairs with longer axes. Probably, the same regulatory mechanism results in the stronger CO covariation in human females as they have a longer axis than males.

### Chromosome axis length regulates CO covariation

Various results support that CO frequency is mainly regulated by chromosome axis length (introduction). Here, we show that chromosome axis length regulates CO covariation in two ways. First, long-axis chromosomes show strong axis length covariations (Figures 5A and 5B), and as a result, they directly generate strong CO correlations. Second, a long-axis chromosome has a strong correlation between axis length and CO number (Figure 5E), which also contributes to the strong CO correlation.

A chromosome usually obtains at least one CO, the obligatory CO, regardless of its axis length (Jones and Franklin, 2006; Zhang et al., 2014a). The maximal number of COs on a chromosome is largely determined by the interplay between the axis length and CO interference distance (Wang et al., 2021a; Shang et al., 2022). When the axis length is less than the interference distance, that is, interference can spread outward to both ends of the chromosome, this chromosome can get only one CO. When the axis length is longer than the interference distance, this chromosome can get more than one CO. Thus, for the longer chromosome axis, the maximal number of COs is less affected by the obligatory CO. The maximal number of COs is also constrained by the effects of centromeres, chromosome ends, and maybe unknown factors (e.g., Zhang et al., 2014a; Wang et al., 2021a). As CO interference seems to be maintained constantly (in terms of microns axis length) among chromosomes and between males and females (Wang et al., 2017, 2019c, 2021a), long-axis chromosomes obtain more COs and are less affected by the above effects. Additionally, with the same degree of variation in axis length, chromosomes with long axes have larger variations in absolute axis length (e.g., alterations in absolute axis length on long chromosomes could be larger than CO interference distance), which would cause corresponding alterations in CO number. In contrast, chromosomes with short axes have smaller variations in absolute axis length, which would less likely cause alterations in CO number. Therefore, the correlation strength between axis length and CO number is stronger on long-axis chromosomes than on short chromosomes. These results further argue for the important roles of chromosome axis length, which can be obtained only from the cytological analysis of meiocytes, in characterizing CO recombination and related features, such as factors regulating CO covariation, CO frequency difference, chromosome loop/axis organization, and CO interference strength (Zhang et al., 2014b; Wang et al., 2017, 2019c; Luo et al., 2019; Lloyd and Jenczewski, 2019).

An interesting phenomenon is that for meiocytes with the same strength of CO correlations, gametes/progenies from nuclei with more COs show stronger CO covariation (Figure 4). Without chromatid interference, a chromatid has only a 50% probability of obtaining a CO from a bivalent, which decreases the CO correlation coefficient in gametes/progenies. Compared with human females, each male bivalent has 1–3 (average 2) COs, and a chromatid can obtain only 0–2 (average 1) COs. This could severely decrease the CO correlation in sperm compared with eggs and further increase the CO correlation difference in gametes between human males and females.

### A new feature of chromosome loop/axis regulation: chromosomes with long axes vary more coordinately within nuclei

Chromosomes with long axes vary more coordinately than chromosomes with short axes within nuclei (Figures 5A and 5B). Therefore, long and short chromosomes are differently organized and regulated within nuclei. The reason and the underlying mechanism are unknown.

There are several well-known examples of different chromosome loop/axis organizations. One example is the different organizations of euchromatin and heterochromatin. Euchromatin is packaged with short loops, and heterochromatin (e.g., rDNA region) is packaged with large loops; thus, euchromatin has less DNA content than heterochromatin per unit length of an axis (e.g., Sherman and Stack, 1995; Peterson et al., 1996; Lhuissier et al., 2007). Another example is that the pseudoautosomal region (PAR) of mammalian males is specifically organized with a super long axis and short loops compared with the genome-wide average to ensure the formation of COs during meiosis (Kauppi et al., 2011; Boekhout et al., 2019; Acquaviva et al., 2020). In budding yeast meiosis, short chromosomes have different protein compositions and are organized to have relatively longer chromosome axes per Mb DNA (Panizza et al., 2011; Murakami et al., 2020). The organization of short chromosomes may be affected more severely by centromeric and telomeric regions, which take up a relatively large part of short chromosomes, and the different protein compositions on short chromosomes may also affect the organization. Variations in any of these factors would affect long and short chromosomes differently.

Meiosis-specific depletion of Pds5, a regulator of the cohesin complex, results in a shorter chromosome axis in several organisms examined (Ding et al., 2006; Jin et al., 2009; Viera et al., 2020). Recently, we have demonstrated that Pds5 regulates the chromosome axis in a dosage-dependent manner in budding yeast meiosis (Song et al., 2021). It seems that the axis lengths of long chromosomes have a larger decrease than short chromosomes when Pds5 is depleted during meiosis (Figure S8). This implies that Pds5 affects chromosome organization differently among chromosomes with different axis lengths. Our most recent study further suggests that Pds5 works as a buffer to antagonizes ubiquitin-associated axis shortening (and thus keep axis length more constantly) by recruiting proteasomes to chromosomes. A loop/extrusion hypothesis has been proposed to explain the chromosome organization in both mitosis and meiosis, during which, cohesin is the possible extruder (Novak et al., 2008; Ding et al., 2016; Fudenberg et al., 2016). Based on this idea, the Pds5-ubiquitin/proteasome pathway may regulate axis length by modulating loop extrusion velocity or the density of barriers that inhibit the looping process. It would be interesting to investigate whether the more coordinated variations of chromosomes with long axes are related to the Pds5 and proteasome abundance.

### Do human females provide more benefits in evolutionary adaption than males?

COs shuffle alleles along chromosomes to create favorable combinations of new alleles and disrupt unfavorable combinations of existing alleles for evolutionary adaption. However, COs may also create unfavorable combinations of new alleles and disrupt adapted combinations of alleles. These two opposite effects have to be well balanced (e.g., Maynard Smith, 1978; Bell, 1982; Otto, 2009; McDonald et al., 2016; Stapley et al., 2017; Veller et al., 2019). Our early study suggests that the CO covariation between chromosomes in a single nucleus has an important role in evolutionary adaption (Wang et al., 2019c). Human females have stronger CO covariation and thus higher fractions of nuclei with hyper- and hypo-COs than males (Figure 1). Moreover, the difference in CO covariation between males and females was further enlarged in gametes/progenies (Figure 3). This suggests that human females may have a more important role in evolutionary adaption.

CO maturation inefficiency preferentially converts chromosomes and oocytes with fewer COs (because of CO covariation) susceptible to chromosome segregation errors (e.g., by creating chromosomes with zero or only distal COs) (Wang et al., 2017, 2019c; Hassold et al., 2021). Even if eggs can be generated from these oocytes and fertilized, most of them would be deselected during development (e.g., due to pregnancy loss) (Hassold et al., 2021).

These properties—more COs, higher frequencies of nuclei with hyper- and hypo-COs, and likely de-selection of hypo-CO eggs—support the argument that females probably have a more important role in human evolutionary adaption to changing environments. This idea is consistent with a previous proposal, which was raised based on female fertility over the reproductive life span (Huseynov et al., 2016; Zelazowski et al., 2017; Gruhn et al., 2019).

### Limitations of the study

In human males, long chromosomes tend to have stronger axis length covariation and a stronger correlation between axis length and CO number. This result in combination with modeling and simulation suggests that the stronger CO covariation in females is probably the consequence of the longer chromosome axis. However, this idea requires further investigation when female data are available for such an analysis.

## STAR★Methods

### Key resources table

**Table:** 

REAGENT or RESOURCE	SOURCE	IDENTIFIER
**Software and algorithms**		

Beam-Film Applications	Zhang et al., 2014a	https://app.box.com/s/hv91q2nrtq0cp9n8iy9m
MATLAB	MathWorks	N/A
GraphPad Prism	GraphPad Software, Inc.	N/A

**Other**		

Data used in this paper	See “data and code availability”	N/A

### Resource availability

#### Lead contact

Further information and requests for resources and reagents should be directed to and will be fulfilled by the lead contact, Liangran Zhang (zhangliangran@sdu.edu.cn).

#### Materials availability

This study did not generate new unique reagents.

#### Software availability

Best-fit simulations of CO patterns were performed by an application written in MATLAB, which is available at https://app.box.com/s/hv91q2nrtq0cp9n8iy9m. The application is developed from the “fill-in-the-holes” model, which is originally called the beam-film model (Kleckner et al., 2004; Zhang et al., 2014a; White et al., 2017).

### Experimental model and subject details

This study does not use any experimental models.

### Method details

#### Correlation coefficient analysis

The correlation coefficient of chromosome axis lengths or CO numbers between two (grouped) chromosomes was calculated by Pearson’s linear correlation coefficient analysis. For two chromosome groups, each group contains half number of all chromosomes if there is an even number of total chromosomes, or one group contains one more chromosome than the other if there is an odd number of chromosomes.

#### Covariance analysis

The covariance is calculated by subtracting the intrinsic variance of all chromosomes from the total variance (variance between nuclei), i.e., [covariance] = [total variance] - [intrinsic variance]. The normalized covariance or the contribution of covariance is calculated as the ratio of covariance to total variance, i.e., [covariance contribution] = [covariance]/[total variance].

In the absence of chromatid interference, each gamete obtains half of the number of COs from meiocytes. According to our previous calculation (Wang et al., 2019c), the following relationship exists between CO variances in meiocytes and CO variances in gametes/progenies: [total variance]_g_ = [intrinsic variance]_m_/4 + X/4 + [covariance]_m_/4. In this equation, X represents the average number of COs per meiocyte, subscript g represents “gamete/progeny”, and subscript m represents “meiocytes”. The relationship for the contribution of CO covariances in meiocytes and gametes is [covariance contribution]_m_/[covariance contribution]_g_ = 1 + X/[total variance]_m_. Therefore, the contribution of covariance in gametes is always lower than that in meiocytes, as observed.

As we have shown (Wang et al., 2019c), in “X/[total variance]_m_”, X contributes to the intrinsic CO variance in gametes by binomial sampling and thus decreases the effect of covariance contribution. However, [total variance]_m_ is determined by CO covariance in meiocytes when the intrinsic variance is fixed. The reduction in the covariance contribution is determined by X/[total variance]_m_, which can be rewritten as 1/(X^.^CV^2^). From this expression, both the average number of COs (X) and the coefficient of variation (CV) are larger in human females than in males. Therefore, the reduction in covariance contribution from meiocytes to gametes is smaller in human females than in males.

#### Variability index analysis

Because of the covariation in COs among chromosomes within nuclei, the total number of COs per nucleus varies much more broadly than predicted when COs are regulated independently across chromosomes within nuclei. This covariation concomitantly generates more nuclei with hyper- and hypo-COs. The distributions of the number of nuclei with and without CO covariation are well fitted by normal distributions. The fractions of nuclei with hyper- and hypo-COs can be easily calculated by comparing the two best-fit normal distribution curves or by comparing the distributions of the two data sets (with and without CO covariation). The sum of the fractions of nuclei with hyper- and hypo-COs is variability index. In this study, variability index is calculated based on the two best-fit normal distribution curves. The variability index for axis length can be calculated analogously.

#### Adding type II COs to MLH1 COs to mimic total COs

In humans like many other organisms, there are two types of COs. MLH1 foci label interference-sensitive COs, i.e. Type I COs. COs from DNA sequencing based on nucleotide polymorphisms represent total COs, i.e. the sum of Type I (interference-sensitive) and Type II (interference-insensitive) COs. It has been widely accepted that Type I COs are patterned by interference and Type II COs are randomly distributed on chromosomes. In humans, Type II COs take up ∼10% of total COs (Housworth and Stahl, 2003; Hou et al., 2013; Campbell et al., 2015). To generate the total CO data set from MLH1 CO data and compare CO covariation between MLH1 COs and total COs, ∼10% of COs are randomly put on each chromosome.

#### Prediction of COs in gametes from meiocytes

During meiotic recombination, a CO that is formed between two non-sister (homolog) chromatids may interfere with the probability of another CO on the same pair of homologs to use any of two non-sister chromatids in the same nucleus. This is traditionally called chromatid interference. However, studies argue against the existence of chromatid interference (e.g. Zhao et al., 1995; Hou et al., 2013). Therefore, statistically, each CO in meiocytes will be randomly allocated to any two homologous chromatids and consequently gametes. Based on this, the CO patterns on each chromosome in gametes can be predicted from the CO patterns in meiocytes.

#### Sorting bivalents into nuclei

For each experimental or simulated chromosome, the bivalents from all nuclei were pooled and sorted from the smallest to the largest according to CO numbers or chromosome axis lengths. To obtain the set of “in silico” nuclei with the maximal correlation coefficient, one bivalent was picked orderly from each of the above-sorted chromosome pools and put into a pseudo nucleus. To create a series of “in silico” nucleus sets with gradually decreased correlation strength, a fraction of chromosomes from sorted chromosome pools was randomly picked and randomized, and then bivalents from those chromosome pools were put into nuclei. If all bivalents from all chromosome pools were randomized, all bivalents were completely independent of each other in this “in silico” nucleus set. Note that this sorting does not alter the degree of intrinsic variation across nuclei for a given chromosome. The relationship between the CO correlation and CO covariance and variability index can be determined in each set of nuclei.

#### Best-fit simulations for CO patterns

The simulated CO data used in this study were obtained from previous work, which was done by an application written in MATLAB developed based on the “fill-in-the-holes” model (Wang et al., 2019c).

The “fill-in-the-holes” model is also called the beam-film model, which is proposed to explain the process of CO occurrence and the resultant CO patterns (Kleckner et al., 2004; Zhang et al., 2014a). In this model, there is an array of CO precursors on each bivalent in each nucleus. The CO designation driving force designates the most sensitive precursor as the first CO (the obligatory CO). Upon designation, an interference signal automatically occurs from the designation site and spreads out along the chromosome to inhibit/decrease the probability of the next CO (designation) occurrence. If the next CO designation occurs, it would occur far away from the existing ones because the interference signal “pushes” them away. Finally, two or more CO designations and thus COs stay far away from each other on the same bivalent, which is the well-known phenomenon of CO interference. When the number of precursors changes, the number of CO designations and COs may change correspondingly. However, it will change less proportionally because of the existence of CO interference, and the changed level is negatively correlated with the strength of CO interference. This generates the phenomenon of CO homeostasis. Therefore, from this logic, obligatory CO, CO interference, and CO homeostasis arise from a single biological process (Wang et al., 2015).

The corresponding best-fit simulation based on this model requires three types of parameters (Zhang et al., 2014a; White et al., 2017).

(1) CO precursors. This set of parameters is required to specify the position of each CO precursor on each bivalent and includes the following parameters: (i) the average precursor number (N), (ii) the extent to which the number of precursors varies among different nuclei (B), and (iii) the distribution of precursors along each bivalent (E). The first two parameters specify the number of precursors on each bivalent, and the third parameter specifies the position of each precursor on a given bivalent.
(2) CO designation. This set of parameters determines which precursor can be designated to be a CO and includes three parameters: (i) the maximal CO designation driving force (Smax), (ii) the strength or the distance of CO interference spreads (L), and (iii) the sensitivity and distribution of precursor response to the designation driving force (A). Whether a given precursor can be designated depends on the interaction between the local designation driving force (determined by the Smax and interference/inhibition strength at this site) at this precursor site at the designation moment and the sensitivity (response) of that precursor to this designation.
(3) CO maturation efficiency (M). The process of a designated CO site developing into mature CO is called CO maturation, which is a long biochemical process. Normally, each designation site will develop to a CO, i.e., M = 1. However, in human females and some mutants, some designated sites fail to mature as COs, which means decreased maturation efficiency (M < 1), and this can be caused by any defect in the maturation process (e.g. Wang et al., 2017).

For simulations of variations in chromosome axis length, two separate simulations were performed and then mixed to generate a whole population (). The two simulated subpopulations have the same interference distance, measured in microns of axis length, but different axis lengths and correspondingly scaled precursor numbers. To mimic a certain level of axis length correlation, a fraction of simulated chromosomes from each of the two subpopulations was randomized (Wang et al., 2019c).

### Quantification and statistical analysis

Pearson’s linear correlation coefficient analysis was used in this study. The 95% confidence intervals were estimated by the bootstrapping method using MATLAB, and the standard error (SE) was calculated according to the formula SE = ΔCI/3.92.

The distributions of the number of nuclei with and without CO covariation were fitted by normal distributions. The variability index is the total fraction of nuclei with hyper- and hypo-COs, which was calculated from the two best-fit normal distributions. The levels of statistical significance were indicated in figures and figure legends. n.s. (not significant), p ≥ 0.5; ∗, p < 0.05; ∗∗, p < 0.01; ∗∗∗, p < 0.001.

## Data Availability

• Human MLH1 foci in spermatocytes are from Sun et al. (2006) and Lian et al. (2008) (provided by F. Sun); human female COs based on DNA sequencing of egg nuclei and corresponding polar bodies are from Hou et al. (2013) (provided by S. Xie); human sperm COs characterized by DNA sequencing are from Bell et al. (2020); and human progeny COs characterized by microarray genotyping and DNA sequencing are from Halldorsson et al. (2019). All data are provided in Tables S1, S2, and S3 for easy access. • This paper does not report original code. • Any additional information required to reanalyze the data reported in this paper is available from the lead contact upon reasonable request.

## Supplemental information

Document S1. Figures S1–S8

Table S1. Data for chromosome axis lengths and MLH1 foci, related to Figures 1, 2, and 5

Table S2. Sperm COs, related to Figures 3 and 5

Table S3. Progeny COs, related to Figures 3 and 5

## References

[bib1] Acquaviva L., Boekhout M., Karasu M.E., Brick K., Pratto F., Li T., van Overbeek M., Kauppi L., Camerini-Otero R.D., Jasin M., Keeney S. (2020). Ensuring meiotic DNA break formation in the mouse pseudoautosomal region. Nature.

[bib2] Bell A.D., Mello C.J., Nemesh J., Brumbaugh S.A., Wysoker A., McCarroll S.A. (2020). Insights into variation in meiosis from 31,228 human sperm genomes. Nature.

[bib3] Bell G. (1982).

[bib4] Boekhout M., Karasu M.E., Wang J., Acquaviva L., Pratto F., Brick K., Eng D.Y., Xu J., Camerini-Otero R.D., Patel D.J., Keeney S. (2019). REC114 partner ANKRD31 controls number, timing, and location of meiotic DNA breaks. Mol. Cell.

[bib5] Bojko M. (1985). Human meiosis IX. Crossing over and chiasma formation in oocytes. Carlsberg Res. Commun..

[bib6] Broman K.W., Murray J.C., Sheffield V.C., White R.L., Weber J.L. (1998). Comprehensive human genetic maps: individual and sex-specific variation in recombination. Am. J. Hum. Genet..

[bib7] Campbell C.L., Furlotte N.A., Eriksson N., Hinds D., Auton A. (2015). Escape from crossover interference increases with maternal age. Nat. Commun..

[bib8] Cheng E.Y., Hunt P.A., Naluai-Cecchini T.A., Fligner C.L., Fujimoto V.Y., Pasternack T.L., Schwartz J.M., Steinauer J.E., Woodruff T.J., Cherry S.M. (2009). Meiotic recombination in human oocytes. PLoS Genet..

[bib9] Codina-Pascual M., Campillo M., Kraus J., Speicher M.R., Egozcue J., Navarro J., Benet J. (2006). Crossover frequency and synaptonemal complex length: their variability and effects on human male meiosis. Mol. Hum. Reprod..

[bib10] Coop G., Wen X., Ober C., Pritchard J.K., Przeworski M. (2008). Highresolution mapping of crossovers reveals extensive variation in fine-scale recombination patterns among humans. Science.

[bib11] Ding D.Q., Matsuda A., Okamasa K., Nagahama Y., Haraguchi T., Hiraoka Y. (2016). Meiotic cohesin-based chromosome structure is essential for homologous chromosome pairing in Schizosaccharomyces pombe. Chromosoma.

[bib12] Ding D.Q., Sakurai N., Katou Y., Itoh T., Shirahige K., Haraguchi T., Hiraoka Y. (2006). Meiotic cohesins modulate chromosome compaction during meiotic prophase in fission yeast. J. Cell Biol..

[bib72] Fudenberg G., Imakaev M., Lu C., Goloborodko A., Abdennur N., Mirny L.A. (2016). Formation of Chromosomal Domains by Loop Extrusion. Cell Reports.

[bib13] Gray S., Cohen P.E. (2016). Control of meiotic crossovers: from doublestrand break formation to designation. Annu. Rev. Genet..

[bib14] Gruhn J.R., Rubio C., Broman K.W., Hunt P.A., Hassold T. (2013). Cytological studies of human meiosis: sex-specific differences in recombination originate at, or prior to, establishment of double-strand breaks. PLoS One.

[bib15] Gruhn J.R., Zielinska A.P., Shukla V., Blanshard R., Capalbo A., Cimadomo D., Nikiforov D., Chan A.C., Newnham L.J., Vogel I. (2019). Chromosome errors in human eggs shape natural fertility over reproductive life span. Science.

[bib16] Halldorsson B.V., Palsson G., Stefansson O.A., Jonsson H., Hardarson M.T., Eggertsson H.P., Gunnarsson B., Oddsson A., Halldorsson G.H., Zink F. (2019). Characterizing mutagenic effects of recombination through a sequence-level genetic map. Science.

[bib17] Hassold T., Maylor-Hagen H., Wood A., Gruhn J., Hoffmann E., Broman K.W., Hunt P. (2021). Failure to recombine is a common feature of human oogenesis. Am. J. Hum. Genet..

[bib18] Hou Y., Fan W., Yan L., Li R., Lian Y., Huang J., Li J., Xu L., Tang F., Xie X.S., Qiao J. (2013). Genome analyses of single human oocytes. Cell.

[bib19] Housworth E.A., Stahl F.W. (2003). Crossover interference in humans. Am. J. Hum. Genet..

[bib20] Hunter N. (2015). Meiotic recombination: the essence of heredity. Cold Spring Harb. Perspect. Biol..

[bib21] Huseynov A., Zollikofer C.P., Coudyzer W., Gascho D., Kellenberger C., Hinzpeter R., Ponce de León M.S. (2016). Developmental evidence for obstetric adaptation of the human female pelvis. Proc. Natl. Acad. Sci. U S A.

[bib22] Jin H., Guacci V., Yu H.G. (2009). Pds5 is required for homologue pairing and inhibits synapsis of sister chromatids during yeast meiosis. J. Cell Biol..

[bib23] Jones G.H., Franklin F.C. (2006). Meiotic crossing-over: obligation and interference. Cell.

[bib24] Kauppi L., Barchi M., Baudat F., Romanienko P.J., Keeney S., and& Jasin M. (2011). Distinct properties of the XY pseudoautosomal region crucial for male meiosis. Science.

[bib25] Kleckner N. (2006). Chiasma formation: chromatin/axis interplay and the role(s) of the synaptonemal complex. Chromosoma.

[bib26] Kleckner N., Storlazzi A., Zickler D. (2003). Coordinate variation in meiotic pachytene SC length and total crossover/chiasma frequency under conditions of constant DNA length. Trends Genet..

[bib27] Kleckner N., Zickler D., Jones G.H., Dekker J., Padmore R., Henle J., Hutchinson J. (2004). A mechanical basis for chromosome function. Proc. Natl. Acad. Sci. U S A.

[bib28] Kong A., Gudbjartsson D.F., Sainz J., Jonsdottir G.M., Gudjonsson S.A., Richardsson B., Sigurdardottir S., Barnard J., Hallbeck B., Masson G. (2002). A high-resolution recombination map of the human genome. Nat. Genet..

[bib29] Lenzi M.L., Smith J., Snowden T., Kim M., Fishel R., Poulos B.K., Cohen P.E. (2005). Extreme heterogeneity in the molecular events leading to the establishment of chiasmata during meiosis I in human oocytes. Am. J. Hum. Genet..

[bib30] Lhuissier F.G., Offenberg H.H., Wittich P.E., Vischer N.O., Heyting C. (2007). The mismatch repair protein MLH1 marks a subset of strongly interfering crossovers in tomato. Plant Cell.

[bib31] Lian J., Yin Y., Oliver-Bonet M., Liehr T., Ko E., Turek P., Sun F., Martin R.H. (2008). Variation in crossover interference levels on individual chromosomes from human males. Hum. Mol. Genet..

[bib32] Lloyd A., Jenczewski E. (2019). Modelling sex-specific crossover patterning in arabidopsis. Genetics.

[bib33] Luo C., Li X., Zhang Q., Yan J. (2019). Single gametophyte sequencing reveals that crossover events differ between sexes in maize. Nat. Commun..

[bib34] Lynn A., Koehler K.E., Judis L., Chan E.R., Cherry J.P., Schwartz S., Seftel A., Hunt P.A., Hassold T.J. (2002). Covariation of synaptonemal complex length and mammalian meiotic exchange rates. Science.

[bib35] Lynn A., Ashley T., Hassold T. (2004). Variation in human meiotic recombination. Annu. Rev. Genomics Hum. Genet..

[bib37] Maynard Smith J. (1978).

[bib38] McDonald M.J., Rice D.P., Desai M.M. (2016). Sex speeds adaptation by altering the dynamics of molecular evolution. Nature.

[bib39] Murakami H., Lam I., Huang P.C., Song J., van Overbeek M., Keeney S. (2020). Multilayered mechanisms ensure that short chromosomes recombine in meiosis. Nature.

[bib40] Nagaoka S.I., Hassold T.J., Hunt P.A. (2012). Human aneuploidy: mechanisms and new insights into an age-old problem. Nat. Rev. Genet..

[bib41] Novak I., Wang H., Revenkova E., Jessberger R., Scherthan H., Höög C. (2008). Cohesin Smc1beta determines meiotic chromatin axis loop organization. J. Cell Biol..

[bib42] Otto S.P. (2009). The evolutionary enigma of sex. Am. Nat..

[bib43] Ottolini C.S., Newnham L., Capalbo A., Natesan S.A., Joshi H.A., Cimadomo D., Griffin D.K., Sage K., Summers M.C., Thornhill A.R. (2015). Genome-wide maps of recombination and chromosome segregation in human oocytes and embryos show selection for maternal recombination rates. Nat. Genet..

[bib44] Panizza S., Mendoza M.A., Berlinger M., Huang L., Nicolas A., Shirahige K., Klein F. (2011). Spo11-accessory proteins link double-strand break sites to the chromosome axis in early meiotic recombination. Cell.

[bib45] Pazhayam N.M., Turcotte C.A., Sekelsky J. (2021). Meiotic crossover patterning. Front. Cell Dev. Biol..

[bib46] Peterson D.G., Stack S.M., Price J.H., Johnston J.S. (1996). DNA content of heterochromatin and euchromatin in tomato (Lycopersicon esculentum) pachytene chromosomes. Genome.

[bib47] Sandor C., Li W., Coppieters W., Druet T., Charlier C., Georges M. (2012). Genetic variants in REC8, RNF212, and PRDM9 influence male recombination in cattle. PLoS Genet..

[bib48] Shang Y., Tan T., Fan C., Nie H., Wang Y., Yang X., Zhai B., Wang S., Zhang L. (2022). Meiotic chromosome organization and crossover patterns. Bio. Reprod..

[bib49] Sherman J.D., Stack S.M. (1995). Two-dimensional spreads of synaptonemal complexes from solanaceous plants. VI. High-resolution recombination nodule map for tomato (Lycopersicon esculentum). Genetics.

[bib50] Song M., Zhai B., Yang X., Tan T., Wang Y., Yang X., Tan Y., Chu T., Cao Y., Song Y. (2021). Interplay between Pds5 and Rec8 in regulating chromosome axis length and crossover frequency. Sci. Adv..

[bib51] Stapley J., Feulner P.G.D., Johnston S.E., Santure A.W., Smadja C.M. (2017). Recombination: the good, the bad and the variable. Philos. Trans. R. Soc. Lond. B Biol. Sci..

[bib74] Sun F., Oliver-Bonet M., Liehr T., Starke H., Turek P., Ko E., Rademaker A., Martin R.H. (2006). Variation in MLH1 distribution in recombination maps for individual chromosomes from human males. Hum. Mol. Genet..

[bib53] Tease C., Hultén M.A. (2004). Inter-sex variation in synaptonemal complex lengths largely determine the different recombination rates in male and female germ cells. Cytogenet. Genome Res..

[bib54] Veller C., Kleckner N., Nowak M.A. (2019). A rigorous measure of genome-wide genetic shuffling that takes into account crossover positions and Mendel's second law. Proc. Natl. Acad. Sci. U S A.

[bib55] Viera A., Berenguer I., Ruiz-Torres M., Gómez R., Guajardo A., Barbero J.L., Losada A., Suja J.A. (2020). PDS5 proteins regulate the length of axial elements and telomere integrity during male mouse meiosis. EMBO Rep..

[bib56] von Diezmann L., Rog O. (2021). Let's get physical - mechanisms of crossover interference. J. Cell Sci..

[bib57] Vranis N.M., Van der Heijden G.W., Malki S., Bortvin A. (2010). Synaptonemal complex length variation in wild-type male mice. Genes (Basel)..

[bib58] Wang J., Fan H.C., Behr B., Quake S.R. (2012). Genome-wide single-cell analysis of recombination activity and de novo mutation rates in human sperm. Cell.

[bib59] Wang S., Zickler D., Kleckner N., Zhang L. (2015). Meiotic crossover patterns: obligatory crossover, interference and homeostasis in a single process. Cell Cycle.

[bib60] Wang S., Hassold T., Hunt P., White M.A., Zickler D., Kleckner N., Zhang L. (2017). Inefficient crossover maturation underlies elevated aneuploidy in human female meiosis. Cell.

[bib61] Wang R.J., Dumont B.L., Jing P., Payseur B.A. (2019). A first genetic portrait of synaptonemal complex variation. PLoS Genet..

[bib62] Wang S., Liu Y., Shang Y., Zhai B., Yang X., Kleckner N., Zhang L. (2019). Crossover interference, crossover maturation, and human aneuploidy. BioEssays.

[bib63] Wang S., Veller C., Sun F., Ruiz-Herrera A., Shang Y., Liu H., Zickler D., Chen Z., Kleckner N., Zhang L. (2019). Per-nucleus crossover covariation and implications for evolution. Cell.

[bib64] Wang S., Shang Y., Liu Y., Zhai B., Yang X., Zhang L. (2021). Crossover patterns under meiotic chromosome program. Asian J. Androl..

[bib65] Wang Y., Zhai B., Tan T., Yang X., Zhang J., Song M., Tan Y., Yang X., Chu T., Zhang S. (2021). ESA1 regulates meiotic chromosome axis and crossover frequency via acetylating histone H4. Nucleic Acids Res..

[bib66] White M., Wang S., Zhang L., Kleckner N. (2017). Quantitative Modeling and Automated Analysis of Meiotic Recombination. Methods Mol. Biol..

[bib67] Zelazowski M.J., Sandoval M., Paniker L., Hamilton H.M., Han J., Gribbell M.A., Kang R., Cole F. (2017). Age-dependent alterations in meiotic recombination cause chromosome segregation errors in spermatocytes. Cell.

[bib68] Zhang L., Liang Z., Hutchinson J., Kleckner N. (2014). Crossover patterning by the beam-film model: analysis and implications. PLoS Genet..

[bib69] Zhang L., Wang S., Yin S., Hong S., Kim K.P., Kleckner N. (2014). Topoisomerase II mediates meiotic crossover interference. Nature.

[bib70] Zhao H., McPeek M.S., Speed T.P. (1995). Statistical analysis of chromatid interference. Genetics.

[bib71] Ziolkowski P.A., Underwood C.J., Lambing C., Martinez-Garcia M., Lawrence E.J., Ziolkowska L., Griffin C., Choi K., Franklin F.C., Martienssen R.A., Henderson I.R. (2017). Natural variation and dosage of the HEI10 meiotic E3 ligase control *Arabidopsis* crossover recombination. Genes Dev..

