# Supplementary material for: Per-nucleus crossover covariation is regulated by chromosome organization
Source: iScience. 2022 Mar 18;25(4):104115. doi: 10.1016/j.isci.2022.104115 (PMC8980760; doi:10.1016/j.isci.2022.104115)
Supplement: Document S1. Figures S1–S8 [file mmc1.pdf]

iScience, Volume *25*

Supplemental information

Per-nucleus crossover covariation

is regulated by chromosome organization

Cunxian Fan, Xiao Yang, Hui Nie, Shunxin Wang, and Liangran Zhang

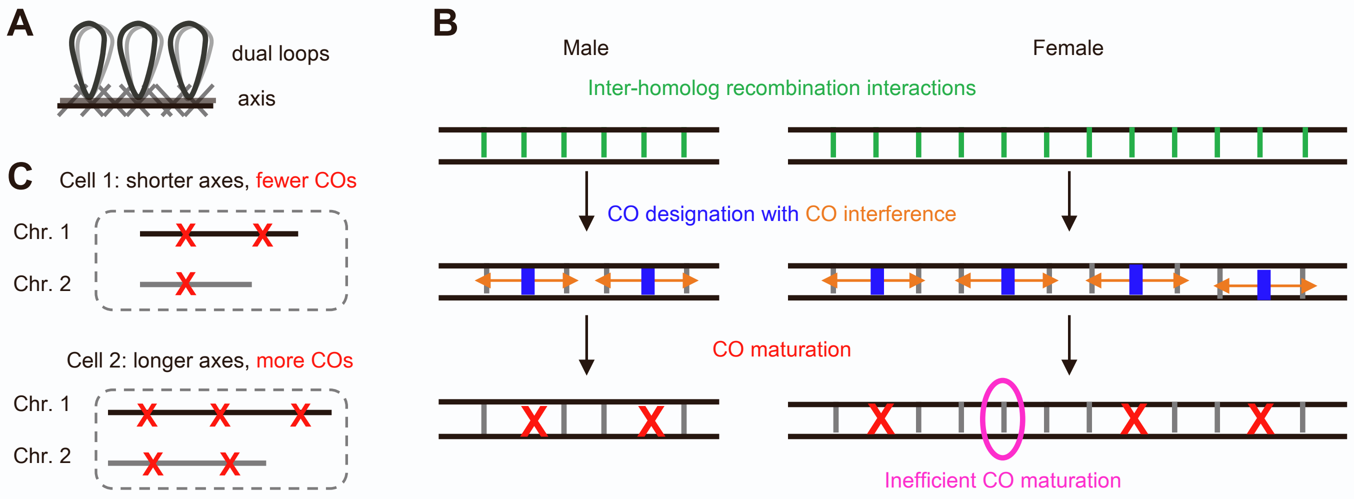

Figure S1. Cartoons for CO covariation and CO recombination differences between human males and females, related to Figure 1.

(**A**) The loop/axis architecture of meiotic chromosomes. (**B**) Human females have longer axes and more recombination intermediates (green bars), and consequently more CO designations (blue bars) and COs (red "X") given same interference strength (orange arrows) in males and females. However, compared to human males, females have CO maturation inefficiency which results in some designated sites failed to develop to actual COs (pink circle). (**C**) If one chromosome has a longer (shorter) axis (black and grey lines), other chromosomes all tend to have longer (shorter) axes within a single nucleus. As a result, each chromosome and thus the nucleus tends to have more (fewer) COs (red "X").

A **Total CO variance = Intrinsic CO variance (variance if independent) + CO covariance**

B

	Decomposition of CO variance			
	**(*A*) = Observed Total Variance**	**(*B*) = Variance if Independent**	**(*C*) = Covariance**	**(*C/A*) = Contribution of Covariance to Total Variance**
**I. COs per nucleus**				
H. male [a]	25.3	6.2	19.1	0.75
H. female [b]	257.0	44.4	212.6	0.83
**II. COs per gamete/individual**				
H. male - sperms [c]	21.5	15.1	6.4	0.29
H. male sperms – pred. from MLH1	18.7	13.9	4.8	0.26
H. Male - progenies [d]				
All ages	19.4	15.1	4.3	0.22
ages <= 20 yrs	18.6	15.0	3.6	0.19
ages 21-40 yrs	19.5	15.1	4.3	0.22
ages >=41 yrs	18.4	15.1	3.2	0.19
H. female - eggs [b]	79.0	28.3	50.7	0.64
H. female eggs – pred. from tetrads	85.4	32.2	53.2	0.62
H. Female - progenies [d]				
All ages	75.9	28.4	47.5	0.63
ages <= 18 yrs	73.4	27.6	45.7	0.62
ages 19-34 yrs	75.7	28.4	48.3	0.63
ages >=35 yrs	76.8	28.9	47.9	0.62

Figure S2. Analysis of CO covariance, related to Figure 1.

(**A**) The total variance of CO number in a cell (*"A"*) is decomposed into the intrinsic variance for individual chromosomes (*"B"*) and the covariance of all chromosomes (*"C"*) (Methods). (**B**) Values of *A*, *B,* and *C* for COs in human meiocytes, gametes, and progenies. The contribution of covariance to total variance is *C/A*. [a]Human spermatocytes (Sun et al., 2006; Lian et al., 2008); [b]Human female "tetrads" (Hou et al., 2013); [c]Human sperm (Bell et al., 2020); [d]Human progenies (Halldorsson et al., 2019).

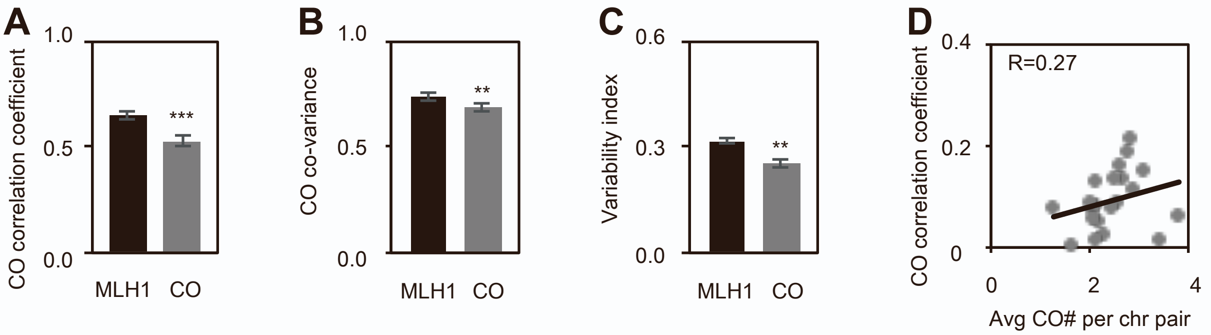

Figure S3. Type II COs decreases the strength of CO covariation, related to Figure 1.

In human, there are two types of COs: Type I or interference-sensitive COs and Type II interference-insensitive COs. MLH1 foci only label Type I COs, which take up ~90% of total COs. COs calculated from DNA sequencing with nucleotide polymorphisms represent total COs, i.e. the sum of Type I and Type II COs. Type II COs are thought to be randomly distributed on chromosomes. To estimate the influence of Type II COs on (Type I) CO covariation, on the basis of human spermatocyte MLH1 data, ~10% of total COs (average 5 per nucleus) were randomly added onto each chromosome in each nucleus (Methods). CO covariation was calculated based on this new CO data set. It is easily imaginable that randomly added Type II COs decrease CO correlation coefficient and thus the strength of CO covariation. (**A**) Total COs show lower CO correlation coefficient than MLH1 labelled COs. (**B**) Total COs show lower CO co-variance than MLH1 labelled COs. (**C**) Total COs show lower variability index than MLH1 labelled COs. (**D**) Correlation coefficient for simulated total COs between adjacent chromosomes plotted against the average CO number, which shows chromosome pairs with more COs tend to have stronger CO correlations. Data are presented as mean ± SE(A-C). ***, $p<0.001$; **, $p<0.01$ by t-test (A-C).

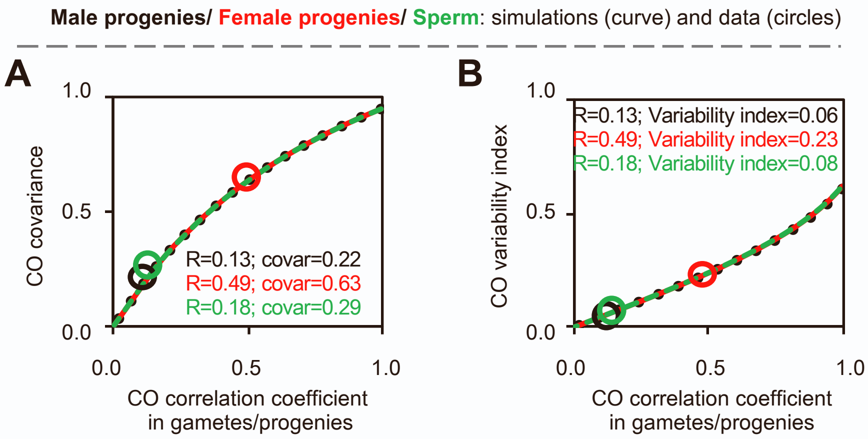

Figure S4. The CO correlation strength determines CO covariation strength and variability index in gametes/progenies, related to Figures 2 and 4.

(**A,B**) As in Figure 2, but simulations based on male progenies (black dot curve), female progenies (red dot curve), and sperm (green dot curve). Black circle, calculated from male progeny data; red circle, calculated from female progeny data; green, calculated from sperm data. The numbers of sperm/eggs and progenies are described in Figures 1 and 3.

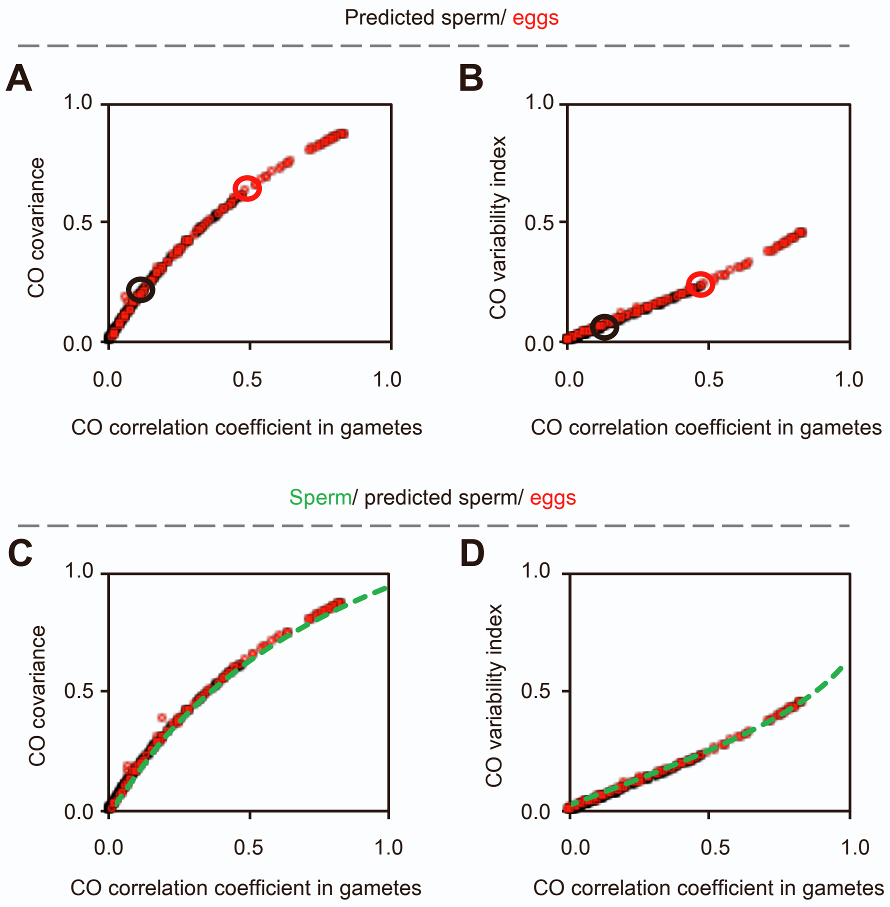

Figure S5. The CO correlation coefficient determines the CO covariance and variability index in gametes/progenies, related to Figure 4.

A series of sets of “in silico” nuclei with different CO correlation coefficients were created based on the sperm, predicted sperm from male MLH1 foci, or eggs, as described in Methods. The CO covariance and variability index were calculated from each set of the “in silico” nuclei. (A,C) CO covariance plotted against the corresponding CO correlation coefficient to generate covariance curves. (B,D) CO variability index plotted against the corresponding CO correlation coefficient to generate variability index curves. The big black circle in (A), calculated from the predicted sperm COs from male MLH1 foci assuming no chromatid interference; the big red circle in (B), calculated from the egg COs.

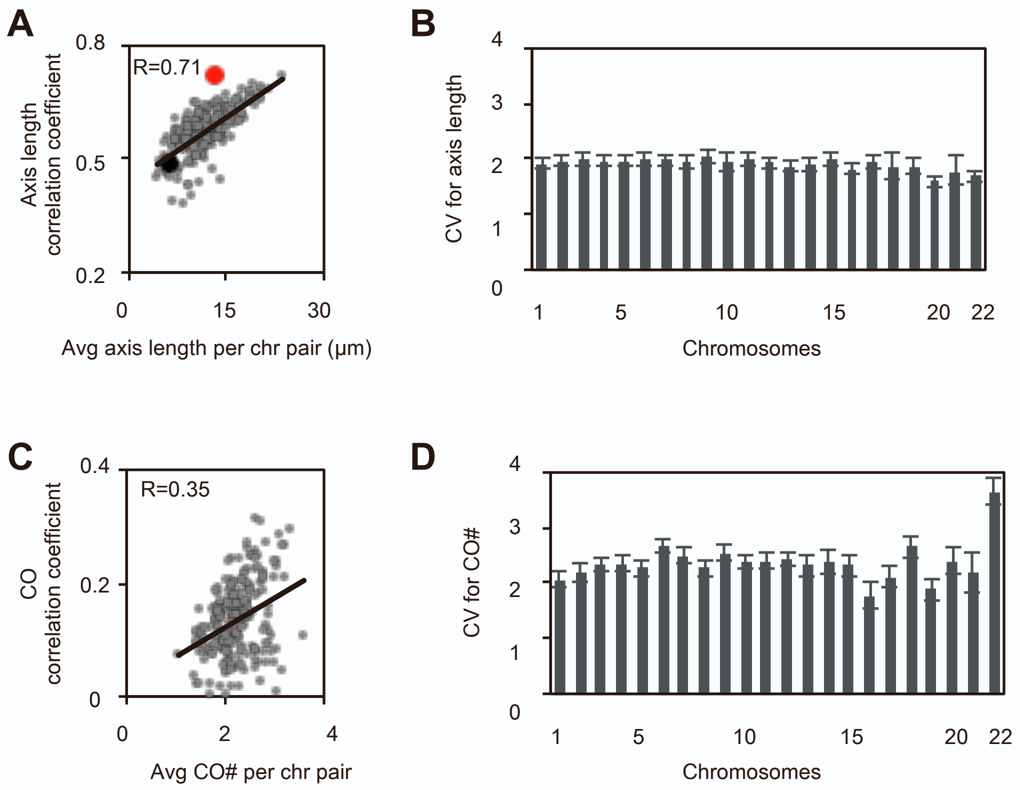

Figure S6. Chromosome pairs with longer axes tend to have stronger axis length correlations and CO correlations, related to Figures 5 and 6.

(**A**) The correlation coefficients of chromosome axis lengths for all chromosome pairs in spermatocytes were plotted against the corresponding average axis length. Chromosome pairs with longer axis lengths tend to have stronger axis length correlations. (**B**) Coefficient of variation (CV) for chromosome axis length in spermatocytes. CV was calculated as the standard deviation divided by the mean. (**C**) CO correlation coefficients from all chromosome pairs in spermatocytes were plotted against the corresponding average CO numbers. Chromosome pairs with more COs tend to have stronger CO correlations. (**D**) CV for CO number in spermatocytes. Data are presented as mean ± 95% confidence interval calculated from bootstrapping of the experimental data (B, D). Sample size, n=755 spermatocytes.

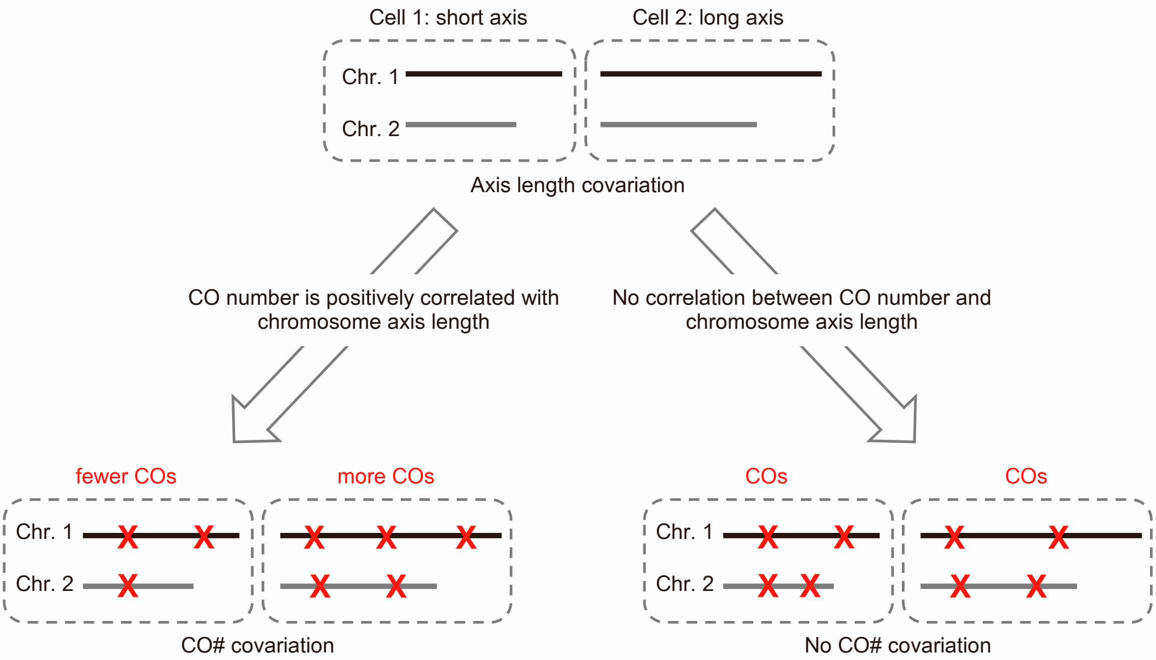

Figure S7. CO covariation requires both axis length covariation and correlation between axis length and CO number, related to Figures 5 and 6.

Chromosome axis length largely determines and consequently is positively correlated with CO number. Therefore, chromosome axis length covariation results in CO covariation. If there is no correlation between axis length and CO number, there is no CO covariation.

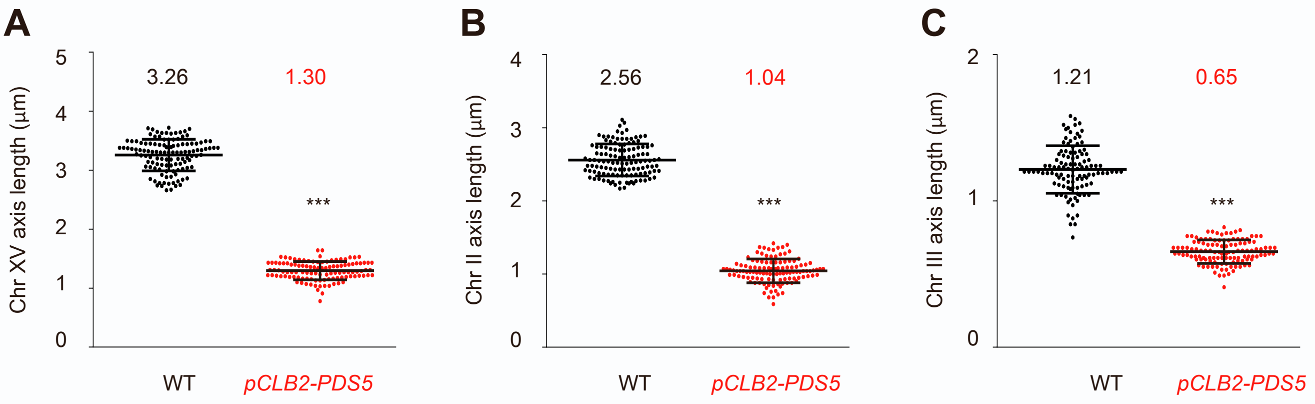

Figure S8. Chromosomes with different axis lengths changed differently in Pds5 depletion in budding yeast meiosis, related to Figures 5 and 6.

The native promoter of Pds5 was replaced by a mitosis-specific CLB2 promoter. (**A,B**) The axis lengths of two long chromosomes (XV and II) were decreased to 40% of WT levels in the *pCLB2-PDS5* mutant. (**C**) The axis lengths of the short chromosome III is decreased to 54% of WT level in the *pCLB2-PDS5* mutant. Data are presented as mean ± SEM (A-C). ***, p<0.001 by t-test (A-C).
